# Hereditary Hemochromatosis Associations with Frailty, Sarcopenia and Chronic Pain: Evidence from 200,975 Older UK Biobank Participants

**DOI:** 10.1093/gerona/gly270

**Published:** 2019-01-16

**Authors:** Jone Tamosauskaite, Janice L Atkins, Luke C Pilling, Chia-Ling Kuo, George A Kuchel, Luigi Ferrucci, David Melzer

**Affiliations:** 1 Epidemiology and Public Health Group, University of Exeter Medical School, UK; 2 Center on Aging, University of Connecticut Health Center, Farmington; 3 Department of Community Medicine and Health Care, Connecticut Institute for Clinical and Translational Science, Institute for Systems Genomics, University of Connecticut Health, Farmington; 4 National Institute on Aging, Baltimore, Maryland

**Keywords:** Muscle, Epidemiology, Genetics, Physical function, UK Biobank

## Abstract

**Background:**

Iron is essential for life but contributes to oxidative damage. In Northern-European ancestry populations, *HFE* gene C282Y mutations are relatively common (0.3%–0.6% rare homozygote prevalence) and associated with excessive iron absorption, fatigue, diabetes, arthritis, and liver disease, especially in men. Iron excess can be prevented or treated but diagnosis is often delayed or missed. Data on sarcopenia, pain, and frailty are scarce.

**Methods:**

Using 200,975 UK Biobank volunteers aged 60–70 years, we tested associations between C282Y homozygosity with Fried frailty, sarcopenia, and chronic pain using logistic regression adjusted for age and technical genetic covariates. As iron overload is progressive (with menstruation protective), we included specific analyses of older (65–70 years) females and males.

**Results:**

One thousand three hundred and twelve (0.65%) participants were C282Y homozygotes; 593 were men (0.62%) and 719 were women (0.68%). C282Y homozygote men had increased likelihoods of reporting chronic pain (odds ratio [OR] 1.23: 95% confidence interval [CI] 1.05–1.45, *p* = .01) and diagnoses of polymyalgia rheumatica, compared to common “wild-type” genotype. They were also more likely to have sarcopenia (OR 2.38: 1.80–3.13, *p* = 9.70 × 10^−10^) and frailty (OR 2.01: 1.45–2.80, *p* = 3.41 × 10^−05^). C282Y homozygote women (*n* = 312, 0.7%) aged 65–70 were more likely to be frail (OR 1.73: 1.05–2.84, *p* = .032) and have chronic knee, hip, and back pain. Overall, 1.50% of frail men and 1.51% of frail women in the 65–70 age group were C282Y homozygous.

**Conclusions:**

*HFE* C282Y homozygosity is associated with substantial excess sarcopenia, frailty, and chronic pain at older ages. Given the availability of treatment, hereditary hemochromatosis is a strong candidate for precision medicine approaches to improve outcomes in late life.

Hereditary hemochromatosis (HH) is characterized by iron accumulation and deposition in parenchymal tissues (1). In European ancestry populations, HH is predominantly caused by an autosomal recessive mutation in the *HFE* gene (C282Y) (2,3), present in 5%–15% of the population, with 0.3%–0.6% homozygote and at highest risk of HH (4). The prevalence of this variant is highest in Northern Europe, especially in Britain and Ireland. The prevalence of C282Y homozygosity is 0.26% in the U.S. population (5), and HH is one of the most common genetic diseases in North America.

Symptoms of C282Y-associated HH usually appear in men aged over 40 years and in postmenopausal women. The typical presentation of clinical HH includes liver cirrhosis, diabetes mellitus, and skin pigmentation (6), although many cases are now identified on biochemical measures (high ferritin and transferrin saturation) in patients presenting with fatigue (6). Some studies have suggested that many individuals with the C282Y genotype do not develop clinical disease (7,8), but a recent review suggested that the lifetime risk of serious liver disease in men is approximately 9% (9). In a study of genotyped patients in the eMERGE network, including clinical biobanks across seven U.S. cohorts (10) (*n* = 106 being C282Y homozygote) survival curve analyses to age 90 suggested that 50% of the males and approximately 25% of the female p.C282Y homozygotes were eventually diagnosed with hemochromatosis (mean age at diagnosis 61.5 years old). The lower penetrance in women is attributed to the iron loss occurring in menstruation and having children (6,11). Phlebotomy provides effective prevention and treatment for many features of HH if started before tissue damage occurs (12), but population screening for HH is not encouraged, and many sufferers are diagnosed late in the course of the disease, or misdiagnosed (6,13).

Although iron is essential for life, excess intracellular iron is a powerful contributor to oxidative damage, a hallmark process of aging (14). Iron has also been linked to the inflammatory response and to mitochondrial dysfunction, and it accumulates during cellular senescence (15). As a result, iron accumulation upregulates at least three different biological hallmarks of aging (16). Nevertheless, to the best of our knowledge, no population studies of the C282Y HFE mutation for associations with common geriatric syndromes have yet been done.

In the current study, we aimed to estimate C282Y associations with sarcopenia, frailty, and chronic pain, in older people, aged 60–70 years, from UK Biobank.

## Methods

### Introducing Cohort

Data used in this cross-sectional study are from the UK Biobank—a large community-based volunteer cohort of 502,642 people aged 40–70 years, recruited through 22 assessment centers across England, Wales, and Scotland. For this analysis, we included all subjects of European descent aged 60 to 70 years at baseline with genotyping data (*n* = 200,975) (17). At baseline, demographics, lifestyle, disease history, and physiological measurements were recorded. Informed consent was given for genotyping, and data linkage to medical records. Prevalent HH diagnoses were ascertained by participants self-reports on the baseline questionnaire, plus ICD-10 coded inpatient hospital records from 1996 to baseline interview. There is a selection bias in this data set as UK Biobank volunteers tended to be healthier at baseline than the general UK population (18). Participants were notified of health-related findings at baseline, but not the results of genotyping. Ethical approval for UK Biobank was obtained from the North West Multi-Centre Research Ethics Committee.

### Genotyping

Genotyping and imputation were performed by the UK Biobank team (19). Briefly, two custom Affymetrix microarrays (>95 identical) directly genotyped over 800,000 genetic variants in 488,377 of the UK Biobank participants. Genotype imputation was successfully performed in 487,442 participants, using the Haplotype Reference Consortium panel to infer nearly 40 million genotypes for genetic variants not directly measured, including the *HFE* variant C282Y (rs1800562). We previously identified 451,243 of these participants as being of European descent (20) who were taken forward for analysis.

### Outcome Ascertainment

We tested four main outcomes: chronic pain, polymyalgia rheumatica, Fried definition frailty, and sarcopenia, as these are some of the main geriatric syndromes affecting the elderly population. Additional outcomes we tested in exploratory analysis included five individual frailty components and five specific sites of chronic pain.

Chronic pain was defined as participants reporting site-specific pain that lasted for more than 3 months (yes/no response) in one or more sites (hip, knee, headache, back, and neck/shoulder pain). Prevalent polymyalgia rheumatica was based on self-reported diagnoses on the baseline questionnaire and ICD-10 coded inpatient hospital records from 1996 to baseline interview.

We modified the Fried Frailty phenotype definition (21) according to the data available in UK Biobank. Our definition of Fried Frailty was presence of three or more of: self-reported weight loss, self-reported exhaustion, self-reported slow walking pace, self-reported low physical activity (lowest 20%, sex-specific), and low measured grip strength (lowest 20%, sex-specific). For weight loss, the question was “Compared with one year ago, has your weight changed?” Response “No” and “Yes – gained weight” coded as no weight loss. Response “Yes – weight loss” coded as yes. For the exhaustion measure, the question was “Over the past two weeks, how often have you felt tired or had little energy?” and responses “more than half the days,” “nearly every day” were coded as “Yes.” For the slow walking pace measure participants were asked “How would you describe your usual walking pace?” with the options “slow pace,” “steady average pace,” “brisk pace.” Response “slow pace” was used in the frailty phenotype. For these self-reported measures, responses “do not know” and “prefer not to answer” were excluded. Participants reported frequency and duration of walking, moderate activity, and vigorous activity based on the validated International Physical Activity Questionnaire (22). Total metabolic equivalent (MET) minutes of exercise per week were calculated and categorized into sex-specific quintiles of physical activity. Grip strength was measured in both hands using a hydraulic hand dynamometer and the maximum reading was used. For the Fried frailty phenotype, participants were only included in analyses if they had data on all five individual components (*n* = 172,384/200,975) (see Supplementary Table 1).

Sarcopenia was defined using the European Working Group on Sarcopenia in Older People (EWGSOP) definition, encompassing measures of low muscle mass (measured by bioimpedance using a Tanita BC 418ma body composition analyser) and low muscle strength (measured by grip strength as described above) (23). Low skeletal muscle mass index, as defined by Janssen (24), was defined as <8.87 kg/m^2^ in males and <6.42 kg/m^2^ in females. Low grip strength was defined as <30 kg in males and <20 kg in females (23).

### Statistical Analysis

We tested baseline associations between C282Y homozygosity and outcomes using logistic regression models. These were adjusted for covariates including population substructure using the first five principal components generated in Northern European descent participants, genotyping microarray (Affymetrix Axiom array 90% participants, Affymetrix BiLEVE array, sharing >95% content). In our overall analysis, we compared C282Y homozygotes against wild type (homozygous common) as the reference group. We analyzed men and women separately. As iron overload is cumulative over time (and therefore with advancing age), but menstruation reduces iron stores, we have included specific analyses of older (65–70 years) female and male groups, the 60–64 year olds, and the whole group together (60–70). We tested C282Y homozygosity status for interactions with daily alcohol intake and smoking status for the four main outcomes, since these environmental exposures may exacerbate C282Y iron overload and clinical outcomes (6,25). We considered a *p* value less than .05 as statistically significant. Analyses used Stata v14.1 (26). Figures were generated in R v3.4.1 using package “metaphor” (v2.0) (27).

## Results

There were 200,975 UK Biobank participants aged 60–70 at baseline with genotype data who could be included in the analyses: of these 1,312 (0.65% or 1 in 153) were C282Y homozygotes; 593 were men and 719 were women (Table 1). Self-reported or hospital-recorded diagnoses of hemochromatosis at baseline were present for 193 participants (0.1% of 200,975). 99.9% of homozygote women between the ages of 60–70 years were postmenopausal (including posthysterectomy), and the mean age at menopause was 49.9 years (*SD* 5.39). The number of participants with missing data on any of the outcomes, by sex, can be found in Supplementary Table 1.

**Table 1.** Description of the Sample of 60–70 Year Old UK Biobank Participants by C282Y Genotype and Sex

	Men 60–70 y Old			Women 60–70 y Old		
Number of C282Y copies	-/-	C282Y/-	C282Y/C282Y	-/-	C282Y/-	C282Y/C282Y
***N* (%**)	80,945 (85.08)	13,599 (14.29)	593 (0.62)	90,214 (85.24)	14,905 (14.08)	719 (0.68)
**Age, mean (*SD***)	64.24 (2.86)	64.19 (2.85)	64.19 (2.84)	64.04 (2.84)	64.02 (2.86)	64.26 (2.82)
**Prevalent Hereditary hemochromatosis diagnosis, *n* (%**)	35 (0.04)	31 (0.23)	81 (13.66)	5 (0.01)	7 (0.05)	34 (4.73)
**Chronic pain, *n* (%**)						
Chronic pain in ≥1 sites	33,987 (41.99)	5,819 (42.79)	283 (47.72)	4,1745 (46.27)	6,960 (46.70)	339 (47.15)
Hip pain 3 mo	7,068 (8.76)	1,267 (9.35)	77 (13.03)	10,939 (12.18)	1,853 (12.49)	105(14.71)
Knee pain 3 mo	15,305 (18.97)	2,606 (19.25)	125 (21.22)	17,115 (19.06)	2,931 (19.77)	154 (21.51)
Back pain 3 mo	13,842 (17.16)	2,385 (17.61)	127 (21.53)	16,805 (18.72)	2,795 (18.86)	150 (21.01)
Neck/shoulder pain 3 mo	12,067 (14.96)	2,028 (14.98)	114 (19.32)	15,589 (17.37)	2,659 (17.93)	115 (16.13)
Headache 3 mo	3,589 (4.45)	557 (4.11)	31 (5.25)	7,174 (8.00)	1,162 (7.85)	52 (7.29)
**Prevalent Polymyalgia rheumatica, *n* (%**)	204 (0.25)	48 (0.35)	5 (0.84)	520 (0.58)	92 (0.62)	1 (0.14)
**Frailty, *n* (%**)						
Frailty (3+ of 5 Fried criteria)	2,572 (3.62)	431 (3.61)	39 (7.65)	2,773 (3.66)	469 (3.75)	28 (4.84)
Weight loss	11,050 (14.02)	1,914 (14.47)	91 (15.72)	13,226 (15.05)	2,159 (14.87)	125 (17.96)
Exhaustion	6,455 (8.32)	1,085 (8.31)	68 (11.93)	8,629 (10.06)	1,365 (9.65)	76 (11.38)
Low grip strength	11,972 (15.00)	2,016 (15.04)	139 (23.68)	12,047 (13.53)	1,990 (13.53)	107 (15.07)
Slow walking speed	8,114 (10.21)	1,370 (10.28)	78 (13.33)	8,493 (9.58)	1,459 (9.96)	70 (9.94)
Low physical activity	14,673 (19.81)	2,379 (19.09)	108 (20.26)	16,298 (20.40)	2,779 (21.05)	129 (20.84)
**Sarcopenia, *n* (%**)	3,341 (4.24)	621 (4.69)	57 (9.95)	11,158 (12.65)	1,893 (13.00)	105 (15.00)
Low muscle mass, *n* (%)	34,629 (43.82)	6,008 (45.28)	279 (48.61)	46,125 (52.11)	7,754 (53.02)	381 (54.12)

### C282Y Associations with Outcomes Adjusted for Technical Covariates

**Figure 1.**
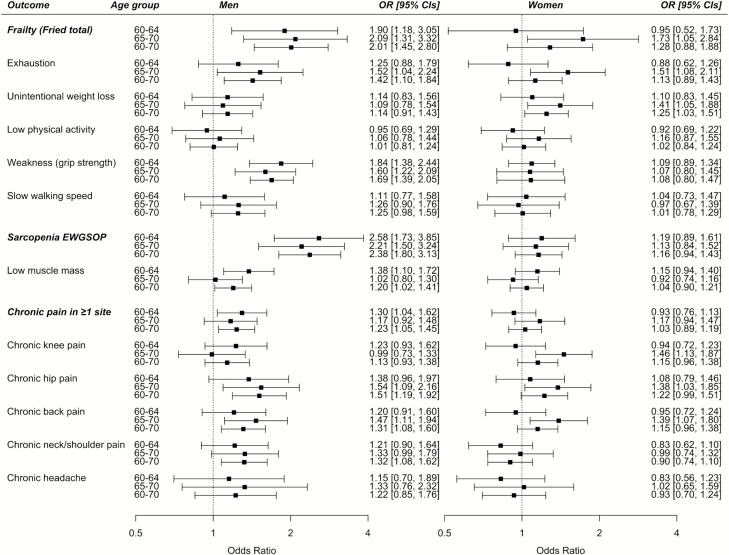
Frailty, sarcopenia, and chronic pain associations with C282Y homozygosity. Forest plot showing odds ratios by health measures comparing C282Y homozygous subjects to those with the common genotype, by sex and age group. Logistic regression models adjusted for age and technical covariates. C282Y homozygote men aged 60–70 years (*n* = 593/95,137); aged 60–64 years (*n* = 315/51,331); and aged 65–70 years (*n* = 278/43,806). C282Y homozygote women aged 60–70 years (*n* = 719/105,838); aged 60–64 years (*n* = 407/60,954); and aged 65–70 years (*n* = 312/44,884). Polymyalgia rheumatica is not in the figure because there were too few observations for the subgroup analyses (see Supplementary Tables 1 and 2).

C282Y homozygote men aged 60–70 years (*n* = 593/95,137) were 2.38 times more likely to have sarcopenia (odds ratio [OR] 2.38, 95% confidence interval [CI] 1.80–3.13, *p* = 9.7 × 10^−10^, compared to the common “wild-type” genotype; 9.95% vs 4.24% in wild type [Table 1, Figure 1]). The association with grip strength (OR 1.69, 95% CI 1.39–2.05, *p* = 9.81 × 10^−08^) was stronger than the association with muscle mass (OR 1.20, 95% CI 1.02–1.41, *p* = .032). C282Y homozygosity was also associated with increased likelihood of frailty (OR 2.01, 95% CI 1.45–2.80, *p* = 3.41 × 10^−05^; 7.7% vs 3.6%). Chronic pain experienced for ≥3 months at ≥1 site (hip, knee, headache, back, and neck/shoulder pain) was more common in homozygous men (OR 1.23, 95% CI 1.05–1.45, *p* = .01; 47.7% of C282Y homozygous men vs 42.0% in wild type) including hip (OR 1.51, 95% CI 1.19–1.92, *p* = .0008), back (OR 1.31, 95% CI 1.08–1.60, *p* = .0072), neck/shoulder pain (OR 1.32, 95% CI 1.08–1.62, *p* = .0079), and diagnosis of polymyalgia rheumatica (OR 3.36, 95% CI 1.37–8.21, *p* = .01) (see Figure 1, Table 1, and Supplementary Table 2).

C282Y homozygote women (*n* = 719/105,838) were not significantly more likely to have sarcopenia or frailty overall across the 60–70 age range. C282Y homozygote women aged 60–64 years (*n* = 407/60,954) did not have excess frailty, but an association was present in C282Y homozygote women aged 65–70 years (*n* = 312/44,884), possibly due to longer exposure to iron overload postmenopause (OR 1.73, 95% CI 1.05–2.84, *p* = .032). Associations were also present for exhaustion (OR 1.51, 95% CI 1.08–2.11, *p* = .016) and unintentional weight loss (OR 1.41, 95% CI 1.05–1.88, *p* = .021). Similar to the men, C282Y homozygote women aged 65–70 years were also more likely to have pain at the hips (OR 1.38, 95% CI 1.03–1.85, *p* = .033), back (OR 1.39, 95% CI 1.07–1.80, *p* = 0.0121), and in contrast to the men, knee pain (OR 1.46, 95% CI 1.13–1.87, *p* = .004) (see Figure 1, Supplementary Table 3)

### Sensitivity Analyses

We repeated analyses for the four main outcomes including an interaction term between C282Y genotype and daily alcohol intake. The only outcome in men or women in which alcohol intake significantly interacted with C282Y homozygosity status was sarcopenia in 65–70-year-old women (int-*p* = .035), with a stronger association observed in daily drinkers. Analyses including an interaction term for current smoking status showed no significant interactions.

In additional sensitivity analyses, we excluded one of each pair of genetically related participants (to the third-degree or closer, 32,736 of 200,975 excluded, 16.3%), to remove familial effects which can inflate genetic associations. In men aged 60–70 years, all C282Y homozygous status associations remained, with the exception of sarcopenia muscle mass definition (OR 1.14, 95% CI 0.95–1.36, *p* = .17), although point estimates were similar (full sample OR 1.20, 95% CI 1.02–1.41, *p* = .032). A nominally significant association with slow walking speed became statistically significant (*p* = .05). In women aged 65–70 years, associations with exhaustion, Fried frailty, chronic knee, and back pain remained (*p* < .05), but the associations with unintentional weight loss and chronic hip pain became nonsignificant (*p* > .05) (Supplementary Table 4)

We tested whether the associations between C282Y and frailty or chronic pain were robust to excluding 193 participants (0.1% of 200,975) with a diagnosis of hemochromatosis (either self-reported or from the hospital admission data at baseline); in men aged 60–70 years, associations with neck/shoulder and hip pain became nonsignificant; and in women aged 65–70 years, associations with chronic hip pain became nonsignificant (*p* > .05, see Supplementary Table 5).

We also carried out additional sensitivity analysis excluding participants with missing data for one or more outcomes (*n* = 31,898/200,975). Results were largely unchanged but in men aged 60–70 years, a nominally significant association with slow walking speed became significant (*p* = .04) but the association with back pain became nonsignificant. In women aged 65–70 years, associations with unintentional weight loss and chronic hip pain became nonsignificant (*p* > .05, see Supplementary Table 6).

## Discussion

Iron is a key contributor for accelerated oxidative damage in aging (28), yet little was known of the effects of the hemochromatosis *HFE* C282Y mutation in older people. In the large UK Biobank volunteer cohort, we found that both male and female older C282Y homozygotes were significantly more likely to have sarcopenia, frailty, and chronic pain, compared to the wild-type (common) variant. They were also more likely to be diagnosed with polymyalgia rheumatica, although numbers of cases were small. Associations with C282Y were more pronounced in men than women, a pattern typical of HH and often attributed to the “natural therapeutic” effect of repeated blood loss with menstruation in premenopausal women (6). However, in the (virtually all postmenopausal) 65–70-year-old women, we observed similar associations to men, with C282Y homozygosity associated with frailty, exhaustion, and hip pain. In addition, women experienced more unintentional weight loss.

We used the Fried Frailty definition, which was derived on a population of noninstitutionalized ≥65-year olds (21) with an estimated 6.9% meeting criteria: this prevalence is somewhat higher than the 4.0% frailty prevalence in the studied UK Biobank community volunteer sample aged 60–70 years at baseline. We used a modified version of the Fried frailty phenotype of which four of the five variables were self-reported; so, there is a small risk of misclassification from reporting bias. However, one study assessing self-reported Fried frailty compared to the measured phenotype actually found it was more discriminatory and predictive of adverse outcomes (29). As far as we are aware, there are no previous studies of sarcopenia or frailty in C282Y homozygotes. Joint pain in HH is typically initially reported in hand joints (proximal interphalangeal and metacarpophalangeal joints) (30), while some studies mention foot and hip joint pain (13). Unfortunately, no data were available on hand pain in UK Biobank. To the best of our knowledge, the association reported here with back pain is novel. The previous literature on HH generally suggests that large joint symptoms are possible but not characteristic of HH, but it is clear from our results that large joint pain is more common in older C282Y homozygotes.

Possible mechanisms for the associations between C282Y homozygosity and excess sarcopenia, frailty, and chronic pain is the deposition of excess iron in widespread parenchymal tissue leading to the production of toxic free radicals with resulting oxidative damage (6,11). Iron has also been linked to the inflammatory response and to mitochondrial dysfunction (15). However, further work is needed to explore specific mechanistic pathways.

This is the largest study to date with HFE genotyping in an older sample. The UK Biobank participants were volunteers, and the sample was enriched for lower behavioral risks and better health status at baseline (18). However, the prevalence of the C282Y variant is similar to previous reports for groups of British and Irish ancestry (2). We have no data on timing of hemochromatosis diagnosis (ie, whether the first diagnosis was from tracing of family members, mild symptoms or after established morbidity, etc.) or receipt of treatment. Data are also not available on iron, ferritin, or transferrin measures. Future work is needed to address the longer term outcomes, especially in women as time from menopause and iron deposition increases. As many people with C282Y-associated HH are currently not diagnosed or diagnosed only after pathology is well established and treatment is likely to be less effective, new clinical approaches are needed to achieve early ascertainment. It is interesting to note that models suggest that targeted population screening for the HFE C282Y homozygous status would be cost effective (31), even before our new findings reported substantial later life sarcopenia, frailty, and chronic pain.

## Conclusion

*HFE* C282Y homozygosity is associated with substantial excess sarcopenia, frailty, and chronic pain at older ages. A higher index of clinical suspicion may be needed in considering this causation in older European descent patients presenting with joint pain, sarcopenia, and frailty. Given the availability of often simple, inexpensive, and benign prevention and treatment, HH is a strong candidate for precision medicine approaches to prevent and manage sarcopenia, frailty, and pain in later life.

## Funding

This work was supported by an award to D.M. by the Medical Research Council (grant number: MR/M023095/1). J.T., L.C.P., and D.M. are supported by the University of Exeter Medical School. J.L.A. is supported by an award to D.M. by the Medical Research Council (grant number: MR/M023095/1). Input from C.-L.K. and G.A.K. was supported by the University of Connecticut Health Center. L.F. is supported by the Intramural Research Program of the National Institute on Aging, U.S. National Institutes of Health.

*Sponsor’s role*: The sponsor had no role in the design of this study, preparation of the paper or the decision to publish.

## Supplementary Material

Supplementary Table 1

Supplementary Table 2

Supplementary Table 3

Supplementary Table 4

Supplementary Table 5

Supplementary Table 6
