# Supplementary material for: Hereditary Hemochromatosis Associations with Frailty, Sarcopenia and Chronic Pain: Evidence from 200,975 Older UK Biobank Participants
Source: J Gerontol A Biol Sci Med Sci. 2019 Jan 16;74(3):337–42. doi: 10.1093/gerona/gly270 (PMC6376086; doi:10.1093/gerona/gly270)
Supplement: Supplementary Table 1 [file gly270_suppl_supplementary-table-1.docx]

**Supplementary Table 1: UK Biobank participants aged 60-70 years with missing data**

|  | Total | | Men | | Women | |
| --- | --- | --- | --- | --- | --- | --- |
|  | **n** | **%** | **n** | **%** | **n** | **%** |
| Total sample | 200,975 | 100 | 95,137 | 47.34 | 105,838 | 52.66 |
| Compete cases (no missing data) | 169,077 | 84.13 | 81,651 | 85.82 | 87,426 | 82.60 |
| Missing data for at least one outcome | 31,898 | 15.87 | 13,486 | 14.18 | 18,412 | 17.40 |
|  |  |  |  |  |  |  |
| Missing data: |  |  |  |  |  |  |
| Frailty (3+ of 5 Fried criteria) | 28,591 | 14.23 | 11,711 | 12.31 | 16,880 | 15.95 |
| Weight loss | 5,218 | 2.60 | 2,504 | 2.63 | 2,714 | 2.56 |
| Exhaustion | 9,167 | 4.56 | 3,906 | 4.11 | 5,261 | 4.97 |
| Low grip strength | 2,692 | 1.34 | 1,311 | 1.38 | 1,381 | 1.30 |
| Slow walking speed | 3,573 | 1.78 | 1,775 | 1.87 | 1,798 | 1.70 |
| Low physical activity | 20,184 | 10.04 | 8,061 | 8.47 | 12,123 | 11.45 |
| Sarcopenia, n (%) | 4,885 | 2.43 | 2,493 | 2.62 | 11.45 | 2.26 |
| Low muscle mass, n (%) | 4,265 | 2.12 | 2,272 | 2.39 | 1,993 | 1.88 |
