# Supplementary material for: Hereditary Hemochromatosis Associations with Frailty, Sarcopenia and Chronic Pain: Evidence from 200,975 Older UK Biobank Participants
Source: J Gerontol A Biol Sci Med Sci. 2019 Jan 16;74(3):337–42. doi: 10.1093/gerona/gly270 (PMC6376086; doi:10.1093/gerona/gly270)
Supplement: Supplementary Table 2 [file gly270_suppl_supplementary-table-2.docx]

**Supplementary Table 2: Outcome associations with C282Y homozygosity in 60-70 year old males**

| Outcome associations with C282Y homozygosity in 60-70 year old males | | | | | |
| --- | --- | --- | --- | --- | --- |
| Variable | Age | Odds ratio | P value | 95% CI lower | 95% CI upper |
|  |  |  |  |  |  |
| Exhaustion | 60-64 | 1.25 | 0.22 | 0.88 | 1.79 |
|  | 65-70 | 1.52 | 0.03 | 1.04 | 2.24 |
|  | 60-70 | 1.42 | 0.01 | 1.10 | 1.84 |
| Unintentional weight loss | 60-64 | 1.14 | 0.43 | 0.83 | 1.56 |
|  | 65-70 | 1.09 | 0.62 | 0.78 | 1.54 |
|  | 60-70 | 1.14 | 0.26 | 0.91 | 1.43 |
| Low physical activity | 60-64 | 0.95 | 0.73 | 0.69 | 1.29 |
|  | 65-70 | 1.06 | 0.70 | 0.78 | 1.44 |
|  | 60-70 | 1.01 | 0.96 | 0.81 | 1.24 |
| Weakness (grip strength) | 60-64 | 1.84 | <0.001 | 1.38 | 2.44 |
|  | 65-70 | 1.60 | <0.001 | 1.22 | 2.09 |
|  | 60-70 | 1.69 | <0.001 | 1.39 | 2.05 |
| Slow walking speed | 60-64 | 1.11 | 0.57 | 0.77 | 1.58 |
|  | 65-70 | 1.26 | 0.19 | 0.90 | 1.76 |
|  | 60-70 | 1.25 | 0.07 | 0.98 | 1.59 |
| Frailty (Fried total) | 60-64 | 1.90 | 0.01 | 1.18 | 3.05 |
|  | 65-70 | 2.09 | 0.002 | 1.31 | 3.32 |
|  | 60-70 | 2.01 | <0.001 | 1.45 | 2.80 |
| Sarcopenia EWGSOP | 60-64 | 2.58 | <0.001 | 1.73 | 3.85 |
|  | 65-70 | 2.21 | <0.001 | 1.50 | 3.24 |
|  | 60-70 | 2.38 | <0.001 | 1.80 | 3.13 |
| Low muscle mass | 60-64 | 1.38 | 0.01 | 1.10 | 1.72 |
|  | 65-70 | 1.02 | 0.86 | 0.80 | 1.30 |
|  | 60-70 | 1.20 | 0.03 | 1.02 | 1.41 |
| Chronic pain in ≥1 site | 60-64 | 1.30 | 0.02 | 1.04 | 1.62 |
|  | 65-70 | 1.17 | 0.19 | 0.92 | 1.48 |
|  | 60-70 | 1.23 | 0.01 | 1.05 | 1.45 |
| Chronic knee pain | 60-64 | 1.23 | 0.15 | 0.93 | 1.62 |
|  | 65-70 | 0.99 | 0.94 | 0.73 | 1.33 |
|  | 60-70 | 1.13 | 0.22 | 0.93 | 1.38 |
| Chronic hip pain | 60-64 | 1.38 | 0.08 | 0.96 | 1.97 |
|  | 65-70 | 1.54 | 0.01 | 1.09 | 2.16 |
|  | 60-70 | 1.51 | <0.001 | 1.19 | 1.92 |
| Chronic back pain | 60-64 | 1.20 | 0.20 | 0.91 | 1.60 |
|  | 65-70 | 1.47 | 0.01 | 1.11 | 1.94 |
|  | 60-70 | 1.31 | 0.01 | 1.08 | 1.60 |
| Chronic neck/shoulder pain | 60-64 | 1.21 | 0.20 | 0.90 | 1.64 |
|  | 65-70 | 1.33 | 0.06 | 0.99 | 1.79 |
|  | 60-70 | 1.32 | 0.01 | 1.08 | 1.62 |
| Chronic headache | 60-64 | 1.15 | 0.57 | 0.70 | 1.89 |
|  | 65-70 | 1.33 | 0.32 | 0.76 | 2.32 |
|  | 60-70 | 1.22 | 0.28 | 0.85 | 1.76 |
| Polymyalgia rheumatica | 60-64 | 5.77 | 0.00 | 1.80 | 18.53 |
|  | 65-70 | 2.04 | 0.32 | 0.50 | 8.32 |
|  | 60-70 | 3.36 | 0.01 | 1.37 | 8.21 |

| Logistic regression models adjusted for age, genotyping array, and PC1-5. | |
| --- | --- |
| rs1800562 genotypes are in comparison to homozygous common (+/+).  C282Y homozygote men aged 60 to 70 years (n=593/95,137); aged 60 to 64 (n=315/51,331) and aged 65 to 70 (n=278/43,806). |  |
