# Supplementary material for: Hereditary Hemochromatosis Associations with Frailty, Sarcopenia and Chronic Pain: Evidence from 200,975 Older UK Biobank Participants
Source: J Gerontol A Biol Sci Med Sci. 2019 Jan 16;74(3):337–42. doi: 10.1093/gerona/gly270 (PMC6376086; doi:10.1093/gerona/gly270)
Supplement: Supplementary Table 3 [file gly270_suppl_supplementary-table-3.docx]

**Supplementary Table 3: Outcome associations with C282Y homozygosity in 60-70 year old females**

| Outcome associations with C282Y homozygosity in 60-70 year old females | | | | | |
| --- | --- | --- | --- | --- | --- |
| Variable | Age | Odds ratio | P value | 95% CI lower | 95% CI upper |
|  |  |  |  |  |  |
| Exhaustion | 60-64 | 0.88 | 0.50 | 0.62 | 1.26 |
|  | 65-70 | 1.51 | 0.02 | 1.08 | 2.11 |
|  | 60-70 | 1.13 | 0.33 | 0.89 | 1.43 |
| Unintentional weight loss | 60-64 | 1.10 | 0.52 | 0.83 | 1.45 |
|  | 65-70 | 1.41 | 0.02 | 1.05 | 1.88 |
|  | 60-70 | 1.25 | 0.03 | 1.03 | 1.51 |
| Low physical activity | 60-64 | 0.92 | 0.57 | 0.69 | 1.22 |
|  | 65-70 | 1.16 | 0.31 | 0.87 | 1.55 |
|  | 60-70 | 1.02 | 0.86 | 0.84 | 1.24 |
| Weakness (grip strength) | 60-64 | 1.09 | 0.41 | 0.89 | 1.34 |
|  | 65-70 | 1.07 | 0.63 | 0.80 | 1.45 |
|  | 60-70 | 1.08 | 0.61 | 0.80 | 1.47 |
| Slow walking speed | 60-64 | 1.04 | 0.83 | 0.73 | 1.47 |
|  | 65-70 | 0.97 | 0.86 | 0.67 | 1.39 |
|  | 60-70 | 1.01 | 0.96 | 0.78 | 1.29 |
| Frailty (Fried total) | 60-64 | 0.95 | 0.86 | 0.52 | 1.73 |
|  | 65-70 | 1.73 | 0.03 | 1.05 | 2.84 |
|  | 60-70 | 1.28 | 0.20 | 0.88 | 1.88 |
| Sarcopenia EWGSOP | 60-64 | 1.19 | 0.25 | 0.89 | 1.61 |
|  | 65-70 | 1.13 | 0.41 | 0.84 | 1.52 |
|  | 60-70 | 1.16 | 0.16 | 0.94 | 1.43 |
| Low muscle mass | 60-64 | 1.15 | 0.17 | 0.94 | 1.40 |
|  | 65-70 | 0.92 | 0.49 | 0.74 | 1.16 |
|  | 60-70 | 1.04 | 0.57 | 0.90 | 1.21 |
| Chronic pain in ≥1 site | 60-64 | 0.93 | 0.47 | 0.76 | 1.13 |
|  | 65-70 | 1.17 | 0.16 | 0.94 | 1.47 |
|  | 60-70 | 1.03 | 0.70 | 0.89 | 1.19 |
| Chronic knee pain | 60-64 | 0.94 | 0.67 | 0.72 | 1.23 |
|  | 65-70 | 1.46 | 0.00 | 1.13 | 1.87 |
|  | 60-70 | 1.15 | 0.12 | 0.96 | 1.38 |
| Chronic hip pain | 60-64 | 1.08 | 0.64 | 0.79 | 1.46 |
|  | 65-70 | 1.38 | 0.03 | 1.03 | 1.85 |
|  | 60-70 | 1.22 | 0.06 | 0.99 | 1.51 |
| Chronic back pain | 60-64 | 0.95 | 0.68 | 0.72 | 1.24 |
|  | 65-70 | 1.39 | 0.01 | 1.07 | 1.80 |
|  | 60-70 | 1.15 | 0.13 | 0.96 | 1.38 |
| Chronic neck/shoulder pain | 60-64 | 0.83 | 0.19 | 0.62 | 1.10 |
|  | 65-70 | 0.99 | 0.93 | 0.74 | 1.32 |
|  | 60-70 | 0.90 | 0.30 | 0.74 | 1.10 |
| Chronic headache | 60-64 | 0.83 | 0.35 | 0.56 | 1.23 |
|  | 65-70 | 1.02 | 0.94 | 0.65 | 1.59 |
|  | 60-70 | 0.93 | 0.62 | 0.70 | 1.24 |
| Polymyalgia rheumatica | 60-64 | 0.57 | 0.58 | 0.08 | 4.08 |
|  | 65-70 | n/a | not enough observations | | |
|  | 60-70 | 0.23 | 0.14 | 0.03 | 1.63 |

| Logistic regression models adjusted for age, genotyping array, and PC1-5. | |
| --- | --- |
| rs1800562 genotypes are in comparison to homozygous common (+/+).  C282Y homozygote women aged 60-70 years (n=719/105,838); aged 60 to 64 (n=407/60,954) and aged 65 to 70 (n=312/44,884). |  |
