# Supplementary material for: Hereditary Hemochromatosis Associations with Frailty, Sarcopenia and Chronic Pain: Evidence from 200,975 Older UK Biobank Participants
Source: J Gerontol A Biol Sci Med Sci. 2019 Jan 16;74(3):337–42. doi: 10.1093/gerona/gly270 (PMC6376086; doi:10.1093/gerona/gly270)
Supplement: Supplementary Table 4 [file gly270_suppl_supplementary-table-4.docx]

**Supplementary Table 4: Outcome associations with C282Y after excluding related participants**

| Outcome associations with C282Y after excluding related participants | | | | | | |
| --- | --- | --- | --- | --- | --- | --- |
|  |  |  |  |  |  |  |
| Variable | **Sex** | **Age** | **Odds ratio** | **P value** | **95% CI lower** | **95% CI upper** |
| Unintentional weight loss | Male | 60-70 | 1.08 | 0.57 | 0.84 | 1.39 |
| Exhaustion | Male | 60-70 | 1.42 | 0.02 | 1.07 | 1.89 |
| Low physical activity | Male | 60-70 | 0.89 | 0.34 | 0.70 | 1.13 |
| Weakness (grip strength) | Male | 60-70 | 1.67 | 0.00 | 1.35 | 2.07 |
| Slow walking speed | Male | 60-70 | 1.31 | 0.05 | 1.00 | 1.70 |
| Frailty (Fried total) | Male | 60-70 | 1.86 | 0.00 | 1.27 | 2.72 |
| Chronic hip pain | Male | 60-70 | 1.46 | 0.01 | 1.12 | 1.92 |
| Chronic knee pain | Male | 60-70 | 1.18 | 0.13 | 0.95 | 1.47 |
| Chronic headache | Male | 60-70 | 1.21 | 0.36 | 0.81 | 1.81 |
| Chronic back pain | Male | 60-70 | 1.34 | 0.01 | 1.08 | 1.67 |
| Chronic neck/shoulder pain | Male | 60-70 | 1.40 | 0.00 | 1.12 | 1.75 |
| Chronic pain in ≥1 site | Male | 60-70 | 1.32 | 0.00 | 1.10 | 1.57 |
| Polymyalgia rheumatica | Male | 60-70 | 3.94 | 0.00 | 1.61 | 9.65 |
| Sarcopenia EWGSOP | Male | 60-70 | 2.30 | 0.00 | 1.68 | 3.14 |
| Low muscle mass | Male | 60-70 | 1.14 | 0.17 | 0.95 | 1.36 |
|  |  |  |  |  |  |  |
| Unintentional weight loss | Female | 65-70 | 1.26 | 0.17 | 0.90 | 1.77 |
| Exhaustion | Female | 65-70 | 1.62 | 0.01 | 1.12 | 2.35 |
| Low physical activity | Female | 65-70 | 1.16 | 0.38 | 0.84 | 1.59 |
| Weakness (grip strength) | Female | 65-70 | 1.07 | 0.68 | 0.77 | 1.50 |
| Slow walking speed | Female | 65-70 | 0.99 | 0.97 | 0.66 | 1.48 |
| Frailty (Fried total) | Female | 65-70 | 2.08 | 0.01 | 1.24 | 3.49 |
| Chronic hip pain | Female | 65-70 | 1.36 | 0.07 | 0.97 | 1.90 |
| Chronic knee pain | Female | 65-70 | 1.45 | 0.01 | 1.09 | 1.92 |
| Chronic headache | Female | 65-70 | 1.10 | 0.70 | 0.68 | 1.78 |
| Chronic back pain | Female | 65-70 | 1.38 | 0.03 | 1.03 | 1.84 |
| Chronic neck/shoulder pain | Female | 65-70 | 1.05 | 0.77 | 0.76 | 1.45 |
| Chronic pain in ≥1 site | Female | 65-70 | 1.13 | 0.33 | 0.88 | 1.45 |
| Polymyalgia rheumatica | Female | 65-70 | n/a | not enough observations | | |
| Sarcopenia EWGSOP | Female | 65-70 | 1.12 | 0.52 | 0.80 | 1.55 |
| Low muscle mass | Female | 65-70 | 0.86 | 0.25 | 0.67 | 1.11 |

| Logistic regression models adjusted for age, genotyping array, and PC1-5. | |
| --- | --- |
| rs1800562 genotypes are in comparison to homozygous common (+/+).  N=168,239 (men: n=80,493; women: n=87,746). |  |
