# Supplementary material for: Hereditary Hemochromatosis Associations with Frailty, Sarcopenia and Chronic Pain: Evidence from 200,975 Older UK Biobank Participants
Source: J Gerontol A Biol Sci Med Sci. 2019 Jan 16;74(3):337–42. doi: 10.1093/gerona/gly270 (PMC6376086; doi:10.1093/gerona/gly270)
Supplement: Supplementary Table 5 [file gly270_suppl_supplementary-table-5.docx]

**Supplementary Table 5: Outcome associations with C282Y after excluding participants with a prevalent Hereditary Hemochromatosis diagnosis**

| Outcome associations with C282Y after excluding participants with a prevalent Hereditary Hemochromatosis diagnosis | | | | | | |
| --- | --- | --- | --- | --- | --- | --- |
|  |  |  |  |  |  |  |
| Variable | **Sex** | **Age** | **Odds ratio** | **P value** | **95% CI lower** | **95% CI upper** |
| Unintentional weight loss | Male | 60-70 | 1.10 | 0.44 | 0.86 | 1.41 |
| Exhaustion | Male | 60-70 | 1.38 | 0.02 | 1.05 | 1.83 |
| Low physical activity | Male | 60-70 | 0.89 | 0.34 | 0.70 | 1.13 |
| Weakness (grip strength) | Male | 60-70 | 1.57 | 0.00 | 1.27 | 1.95 |
| Slow walking speed | Male | 60-70 | 1.06 | 0.66 | 0.81 | 1.40 |
| Frailty (Fried total) | Male | 60-70 | 1.68 | 0.01 | 1.14 | 2.47 |
| Chronic hip pain | Male | 60-70 | 1.22 | 0.16 | 0.92 | 1.62 |
| Chronic knee pain | Male | 60-70 | 1.02 | 0.86 | 0.82 | 1.27 |
| Chronic headache | Male | 60-70 | 1.33 | 0.14 | 0.91 | 1.94 |
| Chronic back pain | Male | 60-70 | 1.24 | 0.05 | 1.00 | 1.54 |
| Chronic neck/shoulder pain | Male | 60-70 | 1.23 | 0.07 | 0.98 | 1.55 |
| Chronic pain in ≥1 site | Male | 60-70 | 1.15 | 0.12 | 0.97 | 1.37 |
| Polymyalgia rheumatica | Male | 60-70 | 3.89 | 0.003 | 1.59 | 9.50 |
| Sarcopenia EWGSOP | Male | 60-70 | 2.06 | <0.001 | 1.50 | 2.82 |
| Low muscle mass | Male | 60-70 | 1.23 | 0.02 | 1.03 | 1.47 |
|  |  |  |  |  |  |  |
| Unintentional weight loss | Female | 65-70 | 1.40 | 0.03 | 1.04 | 1.88 |
| Exhaustion | Female | 65-70 | 1.51 | 0.02 | 1.07 | 2.12 |
| Low physical activity | Female | 65-70 | 1.14 | 0.38 | 0.85 | 1.54 |
| Low physical activity | Female | 65-70 | 1.04 | 0.80 | 0.76 | 1.42 |
| Slow walking speed | Female | 65-70 | 0.96 | 0.82 | 0.66 | 1.39 |
| Frailty (Fried total) | Female | 65-70 | 1.71 | 0.04 | 1.02 | 2.86 |
| Chronic hip pain | Female | 65-70 | 1.30 | 0.09 | 0.96 | 1.77 |
| Chronic knee pain | Female | 65-70 | 1.38 | 0.01 | 1.07 | 1.80 |
| Chronic headache | Female | 65-70 | 1.02 | 0.94 | 0.65 | 1.61 |
| Chronic back pain | Female | 65-70 | 1.38 | 0.02 | 1.06 | 1.80 |
| Chronic neck/shoulder pain | Female | 65-70 | 1.03 | 0.87 | 0.76 | 1.38 |
| Chronic pain in ≥1 site | Female | 65-70 | 1.11 | 0.38 | 0.88 | 1.39 |
| Polymyalgia rheumatica | Female | 65-70 | n/a | not enough observations | | |
| Sarcopenia EWGSOP | Female | 65-70 | 1.10 | 0.54 | 0.81 | 1.49 |
| Low muscle mass | Female | 65-70 | 0.91 | 0.44 | 0.72 | 1.15 |

| Logistic regression models adjusted for age, genotyping array, and PC1-5. | |
| --- | --- |
| rs1800562 genotypes are in comparison to homozygous common (+/+).  N= 200,782 (men: n= 94,990; women: n= 105,792). |  |
