# Supplementary material for: Hereditary Hemochromatosis Associations with Frailty, Sarcopenia and Chronic Pain: Evidence from 200,975 Older UK Biobank Participants
Source: J Gerontol A Biol Sci Med Sci. 2019 Jan 16;74(3):337–42. doi: 10.1093/gerona/gly270 (PMC6376086; doi:10.1093/gerona/gly270)
Supplement: Supplementary Table 6 [file gly270_suppl_supplementary-table-6.docx]

**Supplementary Table 6: Outcome associations with C282Y after excluding participants with missing data for one or more outcomes**

| Variable | Sex | Age | Odds ratio | P value | 95% CI lower | 95% CI upper |
| --- | --- | --- | --- | --- | --- | --- |
| Unintentional weight loss | Male | 60-70 | 1.20 | 0.14 | 0.94 | 1.52 |
| Exhaustion | Male | 60-70 | 1.39 | 0.03 | 1.04 | 1.86 |
| Low physical activity | Male | 60-70 | 1.02 | 0.86 | 0.82 | 1.27 |
| Weakness (grip strength) | Male | 60-70 | 1.60 | <0.001 | 1.28 | 1.99 |
| Slow walking speed | Male | 60-70 | 1.34 | 0.04 | 1.02 | 1.77 |
| Frailty (Fried total) | Male | 60-70 | 2.06 | <0.001 | 1.47 | 2.89 |
| Chronic hip pain | Male | 60-70 | 1.47 | 0.01 | 1.12 | 1.93 |
| Chronic knee pain | Male | 60-70 | 1.16 | 0.18 | 0.93 | 1.45 |
| Chronic headache | Male | 60-70 | 1.33 | 0.15 | 0.90 | 1.96 |
| Chronic back pain | Male | 60-70 | 1.21 | 0.10 | 0.96 | 1.51 |
| Chronic neck/shoulder pain | Male | 60-70 | 1.30 | 0.02 | 1.04 | 1.64 |
| Chronic pain in ≥1 site | Male | 60-70 | 1.22 | 0.03 | 1.02 | 1.46 |
| Polymyalgia rheumatica | Male | 60-70 | 3.39 | 0.02 | 1.25 | 9.20 |
| Sarcopenia EWGSOP | Male | 60-70 | 2.21 | <0.001 | 1.62 | 3.03 |
| Low muscle mass | Male | 60-70 | 1.20 | 0.04 | 1.01 | 1.44 |
|  |  |  |  |  |  |  |
| Unintentional weight loss | Female | 65-70 | 1.32 | 0.10 | 0.95 | 1.84 |
| Exhaustion | Female | 65-70 | 1.61 | 0.01 | 1.11 | 2.34 |
| Low physical activity | Female | 65-70 | 1.19 | 0.25 | 0.88 | 1.62 |
| Weakness (grip strength) | Female | 65-70 | 1.07 | 0.68 | 0.76 | 1.52 |
| Slow walking speed | Female | 65-70 | 0.81 | 0.38 | 0.50 | 1.31 |
| Frailty (Fried total) | Female | 65-70 | 1.69 | 0.04 | 1.01 | 2.82 |
| Chronic hip pain | Female | 65-70 | 1.38 | 0.07 | 0.98 | 1.95 |
| Chronic knee pain | Female | 65-70 | 1.49 | 0.01 | 1.11 | 1.99 |
| Chronic headache | Female | 65-70 | 0.97 | 0.91 | 0.57 | 1.64 |
| Chronic back pain | Female | 65-70 | 1.38 | 0.04 | 1.02 | 1.86 |
| Chronic neck/shoulder pain | Female | 65-70 | 1.00 | 0.99 | 0.71 | 1.40 |
| Chronic pain in ≥1 site | Female | 65-70 | 1.19 | 0.19 | 0.92 | 1.53 |
| Polymyalgia rheumatica | Female | 65-70 | n/a | not enough observations | | |
| Sarcopenia EWGSOP | Female | 65-70 | 1.28 | 0.14 | 0.92 | 1.77 |
| Low muscle mass | Female | 65-70 | 1.11 | 0.45 | 0.85 | 1.44 |

| Logistic regression models adjusted for age, genotyping array, and PC1-5. | |
| --- | --- |
| rs1800562 genotypes are in comparison to homozygous common (+/+).  N=169,077 (men: n=81,651; women: n=87,426). |  |
|  |  |
